# Bacterial feeding, *Leishmania* infection and distinct infection routes induce differential defensin expression in *Lutzomyia longipalpis*

**DOI:** 10.1186/1756-3305-6-12

**Published:** 2013-01-11

**Authors:** Erich L Telleria, Maurício R Viana Sant’Anna, Mohammad O Alkurbi, André N Pitaluga, Rod J Dillon, Yara M Traub-Csekö

**Affiliations:** 1 Laboratório de Biologia Molecular de Parasitos e Vetores, Instituto Oswaldo Cruz-Fiocruz, Rio de Janeiro, RJ, Brazil; 2 School of Health and Medicine, Lancaster University, Lancaster, England, UK; 3 Vector Group, Liverpool School of Tropical Medicine, Liverpool, England, UK; 4 Present address: Yale School of Public Health - LEPH, New Haven, CT, USA

**Keywords:** *Lutzomyia longipalpis*, Defensin, *Leishmania*, Bacteria

## Abstract

**Background:**

Phlebotomine insects harbor bacterial, viral and parasitic pathogens that can cause diseases of public health importance. *Lutzomyia longipalpis* is the main vector of visceral leishmaniasis in the New World. Insects can mount a powerful innate immune response to pathogens. Defensin peptides take part in this response and are known to be active against Gram-positive and Gram-negative bacteria, and some parasites. We studied the expression of a defensin gene from *Lutzomyia longipalpis* to understand its role in sand fly immune response.

**Methods:**

We identified, sequenced and evaluated the expression of a *L*. *longipalpis* defensin gene by semi-quantitative RT-PCR. The gene sequence was compared to other vectors defensins and expression was determined along developmental stages and after exposure of adult female *L*. *longipalpis* to bacteria and *Leishmania*.

**Results:**

Phylogenetic analysis showed that the *L*. *longipalpis* defensin is closely related to a defensin from the Old World sand fly *Phlebotomus duboscqi*. Expression was high in late L4 larvae and pupae in comparison to early larval stages and newly emerged flies. Defensin expression was modulated by oral infection with bacteria. The Gram-positive *Micrococcus luteus* induced early high defensin expression, whilst the Gram-negative entomopathogenic *Serratia marcescens* induced a later response. Bacterial injection also induced defensin expression in adult insects. Female sand flies infected orally with *Leishmania mexicana* showed no significant difference in defensin expression compared to blood fed insects apart from a lower defensin expression 5 days post *Leishmania* infection. When *Leishmania* was introduced into the hemolymph by injection there was no induction of defensin expression until 72 h later.

**Conclusions:**

Our results suggest that *L*. *longipalpis* modulates defensin expression upon bacterial and *Leishmania* infection, with patterns of expression that are distinct among bacterial species and routes of infection.

## Background

Sand flies are vectors of bacterial and parasitic diseases such as bartonellosis and leishmaniasis [1,2]. *Lutzomyia longipalpis* is the main vector of *Leishmania infantum chagasi*, the causative agent of visceral leishmaniasis in South America [2]. Although little is known about sand fly responses to bacterial infection, several studies have focused on molecular events that occur during the establishment of *Leishmania* infection in the insect [3]. *Leishmania* molecules such as lipophosphoglycan (LPG) [4] and chitinase [5] have been shown to contribute to the success of *Leishmania* infections in sand flies. Additionally, sand fly molecules such as galectin receptors [6], digestive proteases [7,8] and a physical barrier such as the peritrophic matrix [9] have been shown to have an important role in *Leishmania* survival within the sand fly gut.

Several studies have described the natural gut microbiota in Old World [10-12] and New World sand flies [13-16] although mechanisms by which sand flies control the microbial balance in the gut are still unknown.

Insects are capable of mounting a complex repertoire of immune responses to maintain gut homeostasis and eliminate pathogens. Cellular responses include phagocytosis by hemocytes and melanotic encapsulation of pathogenic microorganisms through the activation of the phenoloxidase cascade [17]. Humoral responses, on the other hand, lead to the synthesis of a wide range of effector molecules, including antimicrobial peptides (AMPs) [18-20]. AMPs have been described in many insects as having a central role in innate immune responses against bacterial and parasitic infections [21-23]. Among these, defensin (a 4 kDa cationic peptide) has been identified in several insects [23-26] and shown to have a deleterious effect on bacteria [26], *Plasmodium*[27] and *Leishmania*[28,29]. Defensin was shown to be upregulated in *Phlebotomus duboscqi* upon *Leishmania major* infection [29]. Here we identified, sequenced and investigated the expression profile of a *L*. *longipalpis* defensin throughout the sand fly developmental stages, after Gram-positive and Gram-negative bacterial challenges and after sand fly infection with *Leishmania mexicana*.

## Methods

### Defensin gene sequence analysis

Partial *L*. *longipalpis* defensin gene (LlDef1) sequences were obtained from our previous database [30,31] and the full genomic sequence was obtained using primers designed to target the 5'UTR and 3'UTR regions (LlDef1F 5’-TTGGTCATAGCGTGCAGAAG-3’ and LlDef1R 5’-AAAAACATTGAAACATGCGACTT-3’). Sequence identity was determined by similarity using BLAST searches [32] against the NCBI database. Multiple alignments were performed using the MAFFT software [33]. Phylogenetic tree analysis was done using MEGA5 software [34] with Neighbor-Joining test, using the p-distance method with complete deletion and 10,000 replicates for bootstrap value. The molecular model of the *L*. *longipalpis* defensin was built based on the tertiary structure of the *Anopheles gambiae* [PDB:2NY8] [35] and *Phormia terranovae* [PDB:1ICA] [36] peptides present in the Protein Data Bank (PDB) [37]. The defensin sequence of *L*. *longipalpis* and *A*. *gambiae* were deposited on the molecular modeling server of the SWISS-MODEL (Automated Comparative Protein Modeling Server) [38,39] for the creation of a 3D prediction structure. The two structures were visually analyzed using the Swiss PDB Viewer 3.7 [40].

### Insects

All experiments were performed using insects from a laboratory colony of *L*. *longipalpis* established from sand flies caught in Jacobina (Bahia, Brazil) using standard methods [41]. Insects were fed on 70% sucrose *ad libitum* and fed on rabbit blood once a week. The insectary was kept under controlled conditions of temperature (27 ± 1°C), humidity (80–95%), and photoperiod (12 h/12 h). All procedures involving animals were performed in accordance with the UK Government (Home Office), HSE and EC regulations.

### Experimental bacterial feeds

*Escherichia coli* (K12 RM148), *Micrococcus luteus* (A270), *Ochrobactrum* sp. (OM1,198 Jacobina colony isolate), *Pantoea agglomerans* (NCIMB11392), and *Serratia marcescens* (NCIMB 1377) were inoculated on Luria-Bertani (LB) agar plates and incubated overnight for 24 hours at 37°C. Single colonies were transferred to polypropylene tubes, grown overnight in LB liquid medium, centrifuged at 13,200 rpm, re-suspended in 20% sucrose to OD_600_ = 0.2 and offered daily to female *L*. *longipalpis* through cotton wool. All bacteria were viable under these conditions over the duration of the experiment. Bacterial feed experiments were performed in parallel, one for each bacteria species, collecting 3 pools of 3 females each.

### *Leishmania* infections

*Leishmania* infections were performed as previously described [42]. In brief, *L*. *mexicana* (strain MNYC/BZ/62/M379) were cultured at 26°C in M199 medium supplemented with 25 μg/mL gentamicin sulphate (Sigma), 1X BME vitamins (Gibco) and 10% fetal calf serum (PAA). In preparation for infection, 2 mL of heat-inactivated (56°C for 1 hour) rabbit blood was used to re-suspend cultured promastigotes to a final concentration of 2 × 10^6^ promastigotes/mL. Rabbit blood seeded with parasites was offered to *L*. *longipalpis* through chick skin feeders and fully engorged flies were transferred to fresh cages. Sand flies were dissected at 5 days post-infection to confirm successful infections. Control flies were fed on rabbit blood only. The infection experiment was performed once, collecting 3 pools of 3 females each.

### Microinjections

Newly emerged *L*. *longipalpis* were microinjected in the thorax with 18 nL of *E*. *coli* culture in LB medium at OD_600_ = 0.2 or 2 × 10^6^/mL *L*. *mexicana* promastigotes using a Nanoject II microinjector. Control flies were either pricked in the thorax with a borosilicate needle or injected with 18 nL of autoclaved LB medium.

### RNA extractions and RT-PCR

Total RNA was extracted from triplicate samples derived from pools of 3 whole larvae or adult *L*. *longipalpis* using TRI Reagent (Ambion). Semi-quantitative RT-PCR was performed using SuperScript III One-Step RT-PCR Platinum TaqHiFi (Invitrogen) according to manufacturer’s instructions, with 10 ng of template RNA and defensin-specific primers [30] (Defensin F 5’-GCCTGTGTGTTGTGGTTCT-3’; Defensin R 5’- GCATCTCCCCATCCTGTT-3’). Gene transcription was normalized based on the 60S ribosomal protein L3 gene from *L*. *longipalpis* [GenBank: AM088777]. RT-PCR products were resolved on 2% agarose/ethidium bromide gels and band intensity was determined by densitometric measurement using the Image J software [43]. Differential transcription of genes was determined by the ratio between target gene band intensity and the corresponding 60S L3 products obtained from multiplex RT-PCR reactions.

### Statistical analysis

Statistical t-test analysis was performed using the GraphPad Prism software (San Diego, CA, USA). Results were expressed as mean ± SEM. Significance was considered when *P* < 0.05.

## Results

### Defensin gene sequence and phylogeny

**Figure 1:**
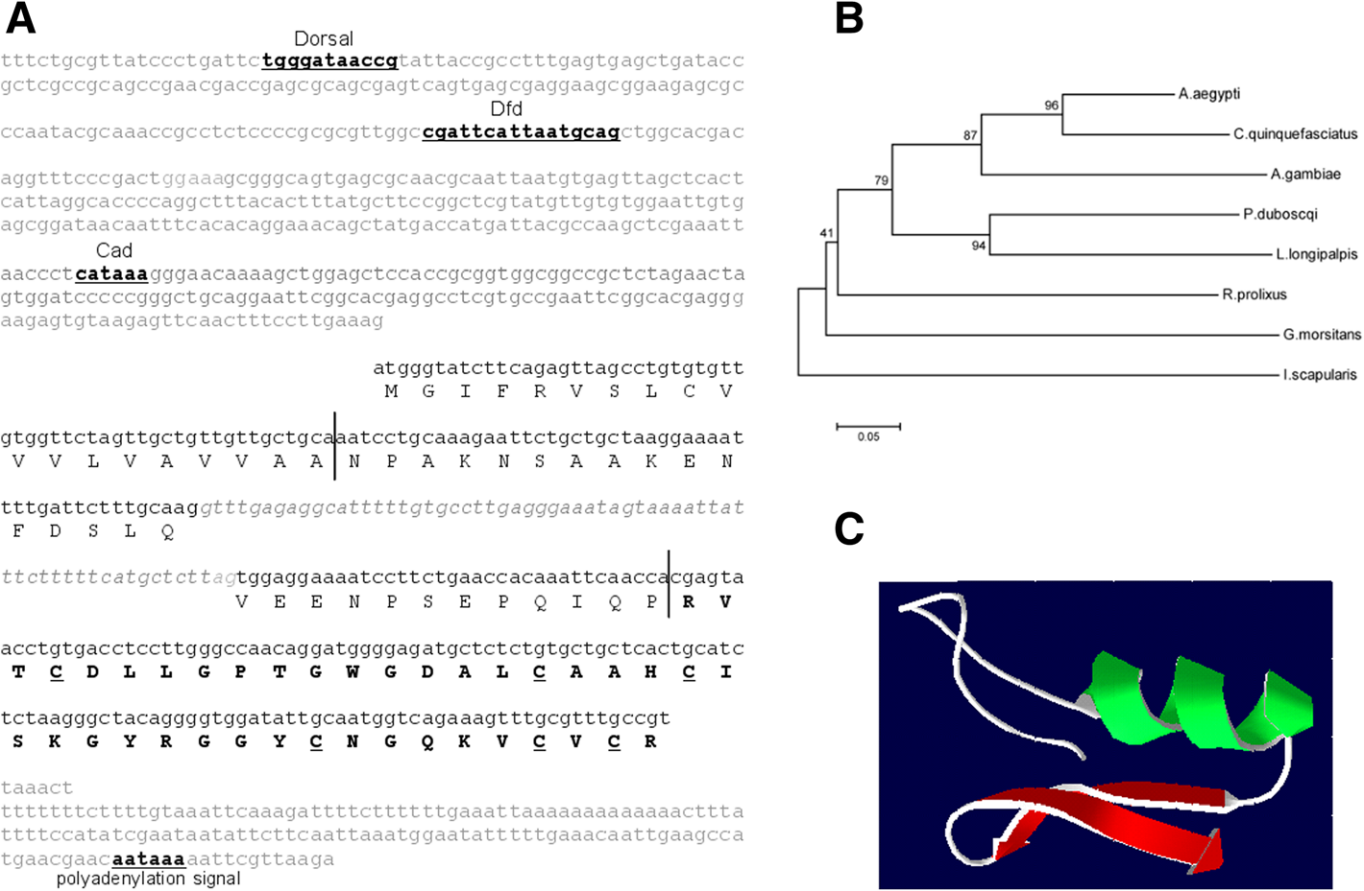
**Sequence, phylogenetic analysis**, **and molecular modeling of *Lutzomyia longipalpis *defensin 1 (LlDef1). **(**A**) The complete genomic sequence of the *Lutzomyia longipalpis *LlDef1 defensin gene containing 1034 nucleotides (nt) is shown. The 5'UTR region contains 518 nt and the 3'UTR 153 nt (lower case gray letters). In the 5'UTR region, potential binding sites for transcription factors Dorsal, Dfd and Caudal are shown (underlined bold lower case letters). The LlDef1 coding region contains 363 nucleotides with a 63 nt intron (gray lower case italic letters). The amino acid prediction indicates an 87 residues peptide (upper case letters), from which 40 correspond to the mature peptide (bold upper case letters). The first vertical bar limits the signal peptide and the second vertical bar divides the pre and pro-peptide. The 6 cysteines of the pro-peptide with the potential to generate 3 disulfide bonds are underlined and the polyadenylation site is indicated. (**B**) Neighbour-joining tree based on multi alignment created from defensins predicted amino acid sequences of *L*. *longipalpis *[JQ970473], *A*. *aegypti *[P81602.2], *A*. *gambiae *[AAC18575.1], *P*. *duboscqi *[P83404.3], *Rhodnius prolixus *[AAO74624.1], *Glossina morsitans *[Q8WTD4.1], *Culex quinquefasciatus *[AEQ27735.1] and *Ixodes scapularis* [XP_002401521.1], showing the phylogenetic relationship between *L*. *longipalpis *and other insect defensins. (**C**) Putative tertiary structure of the *L*. *longipalpis* defensin showing the characteristic architecture of arthropod defensins with two anti-parallel β-sheets (red) and an α-helix (green).

The LlDef1 sequence was shown to contain 1034 nucleotides (nt) with the coding region between nucleotides 512 and 837 and an intron located between nucleotides 617 and 681 (Figure 1A). The 5'UTR sequence displayed putative binding sites for dorsal, caudal and HSF transcription factors and a polyadenylation signal site was found in the 3'UTR. The amino acid prediction indicates an 87 residues peptide, from which 40 correspond to the mature peptide (4.23 kDa) (Figure 1A).

Multiple alignment analysis indicated conserved regions among all defensin sequences selected from blood feeding arthropods (data not shown) and the phylogenetic analysis showed that the *L*. *longipalpis* defensin sequence is closely related to defensins obtained from *P*. *duboscqi* and other nematocerans (Figure 1B). The putative *L*. *longipalpis* defensin tertiary structure was developed based on other insect defensins present in PDB. The analysis of the structure showed the expected architecture with two anti-parallel β-sheets and one α-helix (Figure 1C).

### Transcription of defensin in *L*. *longipalpis* developmental stages

**Figure 2:**
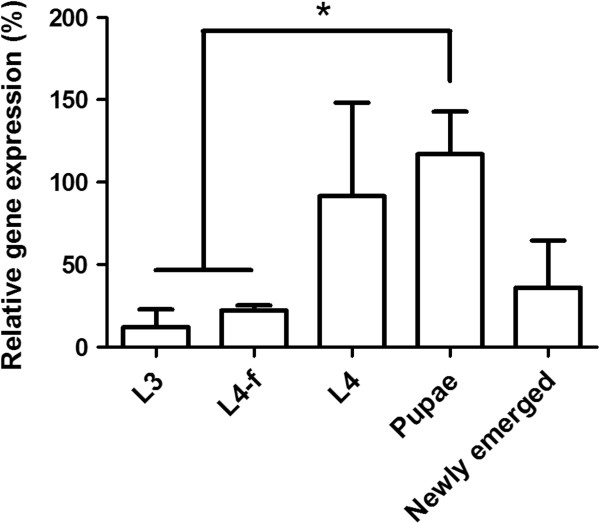
**Defensin expression analysis in *L***. ***longipalpis *developmental stages. **Relative defensin gene expression (normalized against the housekeeping internal control gene 60S rRNA) was determined for feeding larval stages (L3 and L4-f), non-feeding stages (L4 and pupae) and recently emerged female *L*. *longipalpis*. Bar charts represent mean ± SEM of 3 pools of 3 insects. Asterisk indicates statistical significance at *P* < 0.05.

RT-PCR was performed with RNA samples obtained from both immature and newly emerged sand flies. Actively feeding larvae (L3 and L4-f) expressed low defensin levels compared to non-feeding L4 larvae, although the difference was not statistically significant (Figure 2). Defensin expression was significantly lower in L3 and L4-f when compared to pupae. Similarly, newly emerged females showed a trend towards lower levels of defensin expression in comparison to pupae (Figure 2).

### Transcription of defensin in *L*. *longipalpis* fed on bacteria or *Leishmania*

**Figure 3:**
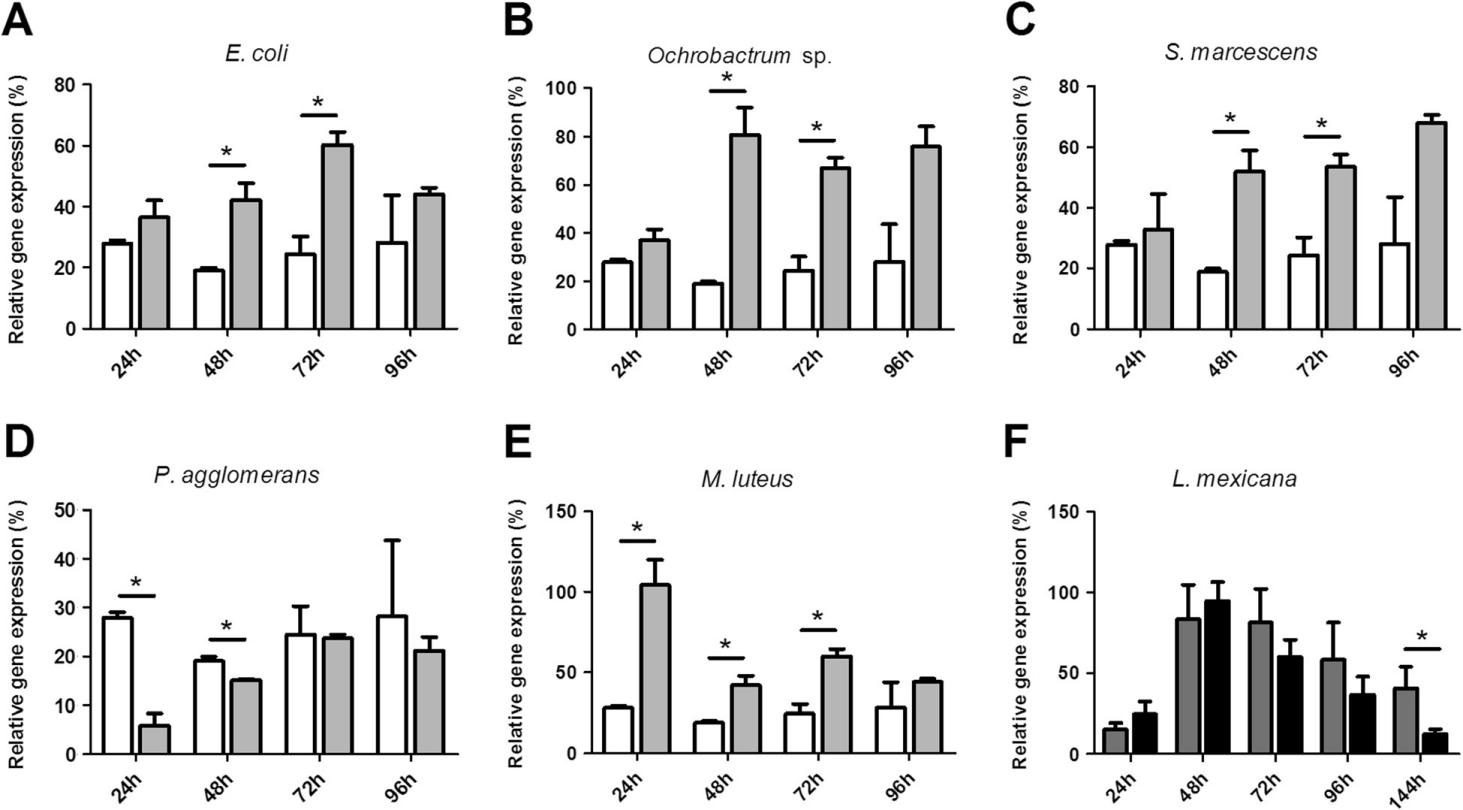
**Defensin expression analysis in *L***. ***longipalpis *fed with bacteria or orally infected with *Leishmania.*** Female *L*. *Longipalpis *fed on suspensions of (**A**) *E*. *coli*; (**B**) *Ochrobactrum *sp.; (**C**) *S*. *marcescens*; (**D**) *P*. *agglomerans*; (**E**) *M*. *luteus*, were collected at 24, 48, 72 and 96 h after feeding (gray bars). Insects fed on sterile sucrose solution were used as control (white bars). (**F**) Female *L*. *longipalpis *fed on blood seeded with *L*. *mexicana *were collected at 24, 48, 72, 96, and 144 h after infection (black bars). Insects fed on blood were used as control (dark gray bars). The relative defensin gene expression was normalized against the housekeeping internal control gene 60S-rRNA. Bar charts represent mean ± SEM of 3 pools of 3 insects. Asterisks represent statistical significance at *P* < 0.05.

Defensin expression increased in female sand flies fed on four out of five bacteria tested when compared to sugar fed controls. This increased defensin expression was statistically significant 48 and 72 h after *E*. *coli*, *Ochrobactrum* sp. or *S*. *marcescens* ingestion (Figure 3A, B and C), and 24, 48 and 72 h after *M*. *luteus* ingestion (Figure 3E). Defensin expression decreased significantly 24 and 48 h after *P*. *agglomerans* ingestion and was unchanged in relation to controls at 72 h (Figure 3D). At the latest time point tested (96 h after infection), defensin expression returned to control levels observed in sugar-fed sand flies for all bacteria tested.

In adult females fed both on blood or blood containing *L*. *mexicana*, defensin expression increased sharply at 48 h and then slowly decreased from that time point until 144 h post-feed. In insects fed on blood containing *Leishmania* defensin expression decreased significantly at 144 h in comparison to blood fed controls (Figure 3F).

**Figure 4:**
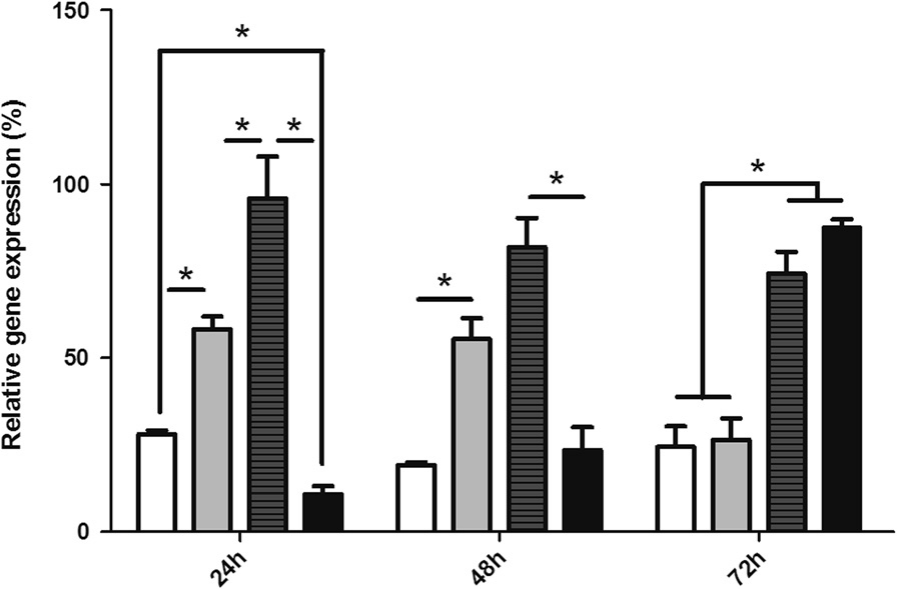
**Defensin expression analysis upon bacterial and *Leishmania *injection into the hemocoel of female *L*. *longipalpis*: Female *L*. *longipalpis *were microinjected with *E*. *coli* (dark gray bars) or *L*. *mexicana *suspensions (black bars) and collected at 24, 48 and 72 h after pathogen inoculation. **Insects fed on sugar (white bars) or injected with LB medium (light gray bars) were used as controls. The relative defensin gene expression was normalized against the housekeeping internal control gene 60S-rRNA. Bar charts represent mean ± SEM of 3 pools of 3 insects. Asterisks represent statistical significance at *P* < 0.05.

Expression of defensin was also investigated in insects exposed to *E*. *coli* and *L*. *mexicana* through injection into the hemocoel. Pricking the insects (data not shown) or injecting female sand flies with autoclaved LB media generated an increase in defensin expression at 24 and 48 h after injection in comparison to uninjected sugar-fed control sand flies (Figure 4). Similarly, female sand flies expressed higher levels of defensin mRNA at 24 and 72 h after *E*. *coli* injection when compared to the mock-injected control group (Figure 4). Insects injected with *L*. *mexicana* initially expressed significantly reduced levels of defensin mRNA at 24 and 48 h after injections, showing increased defensin expression at 72 h after injections when compared to the corresponding control group (Figure 4).

## Discussion

In the present study we investigated and analyzed the expression profile of a defensin gene in *L*. *longipalpis* developmental stages, adult females infected orally with Gram-positive or negative bacteria and *L*. *mexicana*, or injected with *E*. *coli* or *L*. *mexicana*.

The *L*. *longipalpis* LlDef1 defensin gene contains two exons (134 and 172 nt respectively) interspersed with a 63 nt intron. The presence of six cysteines at positions 52, 57, 61, 71, 77 and 79 on the predicted amino acid sequence, with the potential to create three disulfide bonds, characterizes a defensin signature sequence [Pfam 01097]. We also sequenced 511 nt of the LlDef1 5'UTR and the analysis revealed that this gene is potentially under the control of at least two immune-related transcription factors: caudal and dorsal. Caudal encodes a DNA-binding nuclear transcription factor that plays a crucial role during development and innate immune response in *Drosophila*[44]. In *Drosophila*, Dorsal has its nuclear localization enhanced upon microbial challenge, interacting with Pelle, Tube, and Cactus during Toll activation to translocate and bind to NFκB-related sequences of AMP genes inside the nucleus [45]. The phylogenetic analysis showed that LlDef1 is similar to defensin sequences from other nematoceran diptera, being closely related to a *P*. *duboscqi* defensin [29].

High transcription levels were detected in non-feeding *L*. *longipalpis* L4 larvae and pupae. In *Anopheles gambiae*, defensin expression was detected in non-challenged third and fourth instar larvae and pupae, reaching high expression levels after *E*. *coli* injections [46]. A *Drosophila* defensin was detected in third instar larvae only after bacterial challenge, although expression was detected in non-challenged pupae [47], similarly to what was observed in *L*. *longipalpis* and *A*. *gambiae*. No previous study explored the immune response in naturally feeding versus non-feeding larvae in the Diptera group. Since transstadial passage of bacteria from larvae to pupae and adult flies has been already reported for sandflies [12,48,49], *L*. *longipalpis* non-feeding L4 and pupae may trigger defensin expression to control and select gut microbiota during late L4 through pupation to emerged adult.

*L*. *longipalpis* were orally exposed to five different Gram-positive and Gram-negative bacteria. Defensin expression was found to increase with time upon infection with the Gram-negative *E*. *coli*, *Ochrobactrum* sp. and *S*. *marcescens*. *Ochrobactrum* sp. is acquired by *P*. *duboscqi* larvae from the environment [12] and it is plausible to consider that it is recognized by the insect immune system as a foreign antigen as much as *E*. *coli*. *S*. *marcescens* is entomopathogenic and was shown to trigger the *L*. *longipalpis* immune system through ROS increase [50]. Interestingly, infection with the Gram-negative *P*. *agglomerans* showed an initial decrease of defensin expression and a very constant level of expression over time matching control levels. This may be due to the fact that this bacterium, commonly found in *Anopheles stephensi* gut, is not pathogenic [51] and may not be recognized as a hazard by *L*. *longipalpis*. Insect defensins are known to be active mainly against Gram-positive bacteria [23,26,52]. Accordingly, flies exposed to the Gram-positive *M*. *luteus* showed a sharp up-regulation of defensin mRNA during the early stages of infection (24 h post-feeding). Although defensin gene expression dropped considerably during the following 3 days, transcription was still significantly increased at 48 and 72 h post-feeding in comparison to controls.

These results suggest that sand flies are capable of mounting different innate immune responses against distinct bacterial species. A previous study that used the synergistic effects of lysozyme with antibacterial peptides revealed that *L*. *longipalpis* can successfully mount a humoral response against bacterial challenge and this response specifically discriminates between *M*. *luteus* and *E*. *coli*[53]. Although an increase of expression of a 4 kDa peptide was detected in the hemolymph of both *M*. *luteus* and *E*. *coli*-injected *L*. *longipalpis* in comparison to mock-injected controls, an unknown 33 kDa peptide could be detected in the hemolymph of the sand fly only when insects were challenged with *M*. *luteus* but not with *E*. *coli*[53]. These findings, and our present results, suggest that specific and discriminating immune responses are probably produced against the Gram-positive and Gram-negative bacteria in *L*. *longipalpis*.

At 48 h after artificial blood feeding and artificial infection with *L*. *mexicana* adult female sand flies showed a dramatic increase of defensin expression that slowly decreased over time. This initial increase in defensin expression may be a response to the proliferation of sand fly gut microbiota caused by the ingestion of a nutrient-rich blood meal as it was seen in *P*. *duboscqi*[12] and *Aedes aegypti*[54,55]. Interestingly, a defensin down regulation was observed starting at 72 h after *Leishmania* infection, reaching statistical significance at 144 h in comparison to blood-fed controls. Late infections were previously correlated with high numbers of *Leishmania* promastigotes within the sand fly gut [56]. Our present results indicate that high parasite number is correlated to low defensin expression. One explanation of this may be due to low levels of defensin expression at later time points after bloodfeeding, allowing for parasite survival and multiplication. On the other hand, if the defensin expression response is primarily towards bacterial molecular factors then the significant fall in defensin expression may be due to suppression of the gut bacterial population, via a competitive exclusion effect, in the presence of *Leishmania*.

A different transcription profile was reported in *P*. *duboscqi* infected with *Leishmania major*, where low levels of defensin expression were observed in the first day of infection whereas expression was strongly induced at four days after the *Leishmania* infection [29]. It is plausible that different phlebotomine sand flies and different *Leishmania* species may trigger diverse immune responses. This has been reported in mosquitoes, where different immune-related genes were modulated upon infection with various *Plasmodium* species [57,58].

Expression of defensin in *L*. *longipalpis* after *L*. *mexicana* or *E*. *coli* intra-thoracic injection was also investigated. Pricked and LB medium-injected sand flies showed an increase in defensin expression in comparison to uninjected sugar-fed controls at 24 and 48 h post-injection. These results indicate that trauma by injection was sufficient to activate the innate immunity and induce defensin transcription in *L*. *longipalpis*. Cuticle pricking and mock-injection of dsRNA into the sand flies’ hemocoel was shown to reduce the number of *L*. *mexicana* promastigotes within the midgut of *L*. *longipalpis*, possibly by nonspecific activation of the IMD pathway [59]. In *A*. *aegypti*, the injection of sterile saline induced the mosquito immune response and produced low but detectable levels of defensin mRNA [60]. Previous work in *L*. *longipalpis* showed that antimicrobial activity increased in sham-injected insects when compared to non-injected controls [53]. Similarly, our results demonstrated that control *L*. *longipalpis* microinjected with medium showed a significant increase in defensin expression at 24 h in comparison to controls, which was maintained until 48 h post-injection. In *Drosophila* Toll and IMD pathways can regulate different AMPs [61] and both can act synergistically [62]. This much is not yet explored in *L*. *longipalpis*.

Nimmo et al. [53] observed a significant increase in *L*. *longipalpis* humoral response against *E*. *coli* or *M*. *luteus* estimated by inhibition zone assays using hemolymph from bacteria-challenged insects. In addition, *P*. *duboscqi* inoculated with *Erwinia carotovora* showed higher defensin expression in comparison to naive insects and bacteria-fed sand flies [29]. Although in line with results obtained for *P*. *duboscqi*, our results show a much subtler defensin expression in *L*. *longipalpis* upon bacterial injection. Similar results were obtained in *A*. *aegypti* inoculated with *E*. *coli* and *M*. *luteus* which showed 3 times higher levels of defensin peptides in their hemolymph when compared to sterile saline-injected insects [63]. These results confirm that mosquitoes and sand flies can mount an immune response through defensin expression upon bacterial challenge in their hemolymph.

*L*. *longipalpis* injected with *L*. *mexicana* showed a significant increase of defensin expression at 72 h post infection. Although the presence of *Leishmania* in the hemolymph does not occur in nature, it is possible that the ectopic presence of parasites within the hemolymph induced an immune response. It has been shown that *Drosophila* is capable of producing an immune response against injected *Plasmodium gallinaceum* oocytes [64]. Defensin reduction at 24 and 48 h after *Leishmania* injection may be a counterbalance caused by activation of the IMD pathway triggering other AMP, but not DefLl1. Later, at 72 h, *L*. *longipalpis* is able to express high levels of defensin. To our knowledge, this is the first report of an immune response in sandflies after parasite injection. Investigation of other Toll or IMD related AMPs could address and clarify this hypothesis related to the sandflies immune response to *Leishmania* injection in hemolymph, but none has been described up to date.

## Conclusion

Here, we have described a *L*. *longipalpis* defensin gene similar to a *P*. *duboscqi* defensin, modulated by bacterial feed and injection and *Leishmania* infections. These genes are the only defensins so far described for both sand fly species but the presence of multiple defensin genes and other AMPs co-existing in sand flies is possible. Defensin isoforms with distinct transcriptional patterns and putative distinct roles were previously described in *A*. *gambia*e [65]. The difference in defensin expression levels upon bacterial challenge observed for the New and Old World species may therefore be due to expression of different defensin isoforms acting concertedly to control bacterial proliferation within the sand fly midgut and hemolymph. Our results suggest that *L*. *longipalpis* is able to mount a differential response of defensin expression upon bacterial feeding and bacterial injection into the hemocoel and *Leishmania* gut infection.

## Competing interests

The authors declare no competing interests.

## Authors’ contributions

RJD and YMT designed the experiments. ELT and MOA carried out the biological and molecular experiments. ANP performed sequence and phylogenetic analysis. ELT, MRVS, ANP, RJD and YMT wrote the manuscript. All authors read and approved the final version of the manuscript.
